# Age at menarche and endometrial cancer risk: a dose-response meta-analysis of prospective studies

**DOI:** 10.1038/srep14051

**Published:** 2015-09-11

**Authors:** Ting-Ting Gong, Yong-Lai Wang, Xiao-Xin Ma

**Affiliations:** 1 Department of Obstetrics and Gynecology, Shengjing Hospital of China Medical University, Shenyang, China

## Abstract

Evidence between age at menarche and endometrial cancer risk have been controversial. Therefore, we conducted a meta-analysis of prospective studies to analyze the aforementioned association. Relevant studies were identified by searching PubMed and EMBASE databases until the end of June 2015. A random-effects model was used to estimate summary relative risks (RRs) and 95% confidence intervals (CIs) for associations between menarcheal age and endometrial cancer risk. Our meta-analysis included eight prospective studies involving 4553 subjects with endometrial cancer. The summarized RRs of endometrial cancer for menarcheal age were 0.68 (95%CI = 0.58–0.81, *I*^2^ = 41.9%, *P* = 0.099, n = 8) when comparing women with oldest category of menarcheal age with women with youngest category of menarcheal age. Notably, there was an 4% reduction in risk for per 2 years delay in menarcheal age (summarized RR = 0.96; 95%CI = 0.94–0.98, *I*^2^ = 45.7%, *P* = 0.101, n = 6). Additionally, significant inverse associations were consistent within all stratified analyses. There was no evidence of publication bias or significant heterogeneity between subgroups detected by meta-regression analyses. Our findings support the hypothesis that late menarcheal age is inversely associated with endometrial cancer risk. Further larger prospective or pooled studies are warranted to fully adjust for potential confounders and distinguish whether the associations differ by histological subtypes of endometrial cancer.

Endometrial cancer is the second most common gynecologic malignancy worldwide, with approximately 280,000 new cases in 2012^1^. Recently, the development of endometrial cancer may be partly attributed to both endogenous and exogenous of estrogen and progesterone^2^. For example, estrogens are the primary stimulants of endometrial proliferation^3^, which is a prerequisite for carcinogenesis^4^. Apparently, unchecked proliferation can lead to malignant transformation^5^, which follows that estrogens are a cause in the development of at least some endometrial cancer. In contrast, the surge of luteal progesterone secretion which begins just prior to ovulation each month in premenopausal women may play a role in arrest endometrial proliferation and promoting secretory differentiation of the endometrium, which can mitigate against the occurrence of endometrial cancer^2^,^6^,^7^. In 1986, Pettersson *et al.*^8^ first suggested that menstruation span was an important risk factor for endometrial cancer. On the basis of this finding, they addressed that the number of ovulatory cycles might be important in the etiology of this disease^9^. Therefore, reproductive characteristics, such as age at menarche, age at menopause, and nulliparity, that are closely associated with endogenous hormone changes may affect the risk of endometrial cancer^2^.

Menarche was considered to be the milestone of ovulation initiation as well as the initiation of hormone changes in the childhood and adolescent period^10^. Our previous study found a statistically significant inverse association between later menarcheal age and ovarian cancer risk^11^. Furthermore, some studies suggested that increasing rate of overweight/obesity in childhood and adolescent period was inverse associated with the menarcheal age^12^,^13^,^14^. In addition, early menarcheal age increased the risk of all-cause or cardiovascular disease mortality in several studies during the past decade^15^,^16^,^17^,^18^. Although the increased risk of endometrial cancer associated with early menarcheal age has been attributed to a longer lifetime exposure to endogenous estrogen and progesterone deficiency associated with anovulatory cycles^19^,^20^,^21^, the evidence from observational studies are conflicting, which might be partly attributed to different study designs and limited sample sizes of individual studies. Notably, minority of these studies provided the detail information of the risk estimation of per year advance/delay in menarcheal age by dose-response analysis. Therefore, we carried out this meta-analysis of all prospective studies published up to May 2014 to systematically and quantitatively evaluate this issue.

## Results

### Literature search

**Figure 1:**
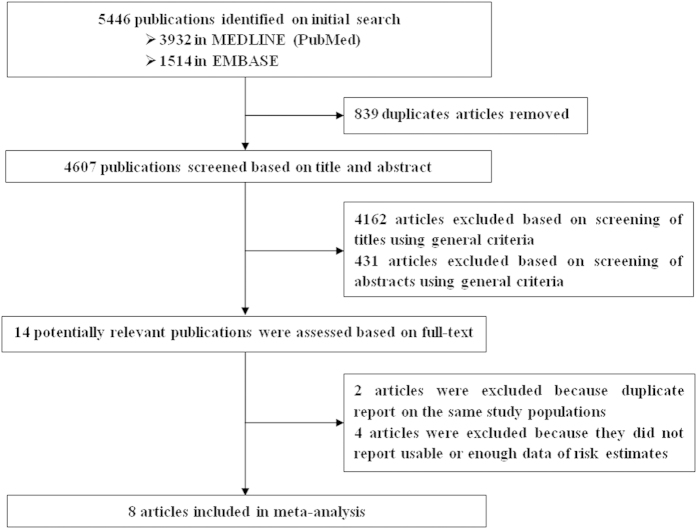
Selection of studies for inclusion in meta-analysis.

We identified 4607 potentially relevant articles from our search of the MEDLINE (PubMed) and EMBASE databases. Of these, 4593 articles were excluded after the first screening based on the abstract or title, leaving 14 articles for full-text review. On this review, two articles^22^,^23^ were excluded because of duplicate reports from the same study population and four articles^24^,^25^,^26^,^27^ were excluded because they did not report sufficient data of risk estimates. Overall, 8 prospective studies were included in this study (Fig. 1).

### Study characteristics

Table 1 summarizes the characteristics of the 8 included studies. These included articles which represent 4,553 cases and 949,945 non-cases, were published between 1988 and 2013, and consist of 7 cohort studies^9^,^21^,^28^,^29^,^30^,^31^,^32^ and one nested case-control study^33^. Of the 8 prospective studies, five were conducted in the United States^28^,^29^,^30^,^31^,^33^, one each in China^9^, Norway^32^, and the European Prospective Investigation into Cancer and Nutrition (EPIC)^21^. Sample sizes of these included studies ranged from 15,528^33^ to 301,601^21^, and the number of endometrial cases varied from 39^33^ to 1450^28^. The median duration of follow-up was 11 years.

### Late *versus* early age at menarche

**Figure 2:**
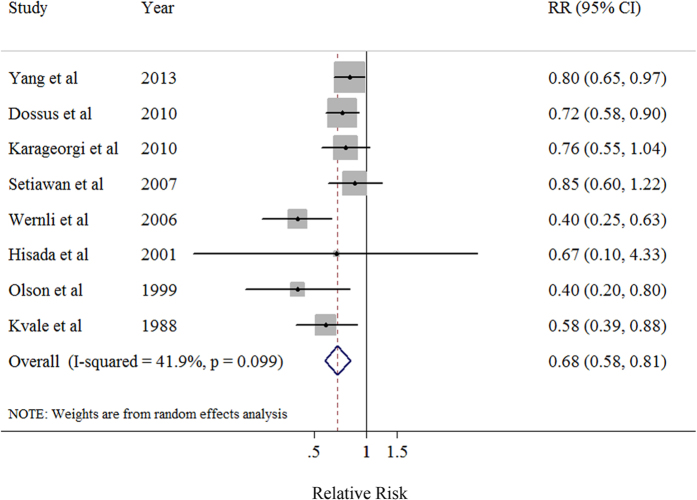
Forest plot (random effects model) of menarcheal age and endometrial cancer risk in prospective studies. Squares indicate study-specific relative risks (size of the square reflects the study-specific statistical weight); horizontal lines indicate 95% CIs; diamond indicates the summary relative risk estimate with its 95% CI. CI: confidence interval; RR: relative risk.

The summary RR of endometrial cancer for the oldest *versus* the youngest categories of menarcheal age was 0.68 (95% CI: 0.58–0.81) with moderate heterogeneity (*I*^2^ = 41.9%, *P* = 0.099) (Table 2, Fig. 2). There was no indication of publication bias with Egger’s test (*P*-bias = 0.189) and no asymmetry in the funnel plots when inspected visually.

### Dose-response analysis

**Figure 3:**
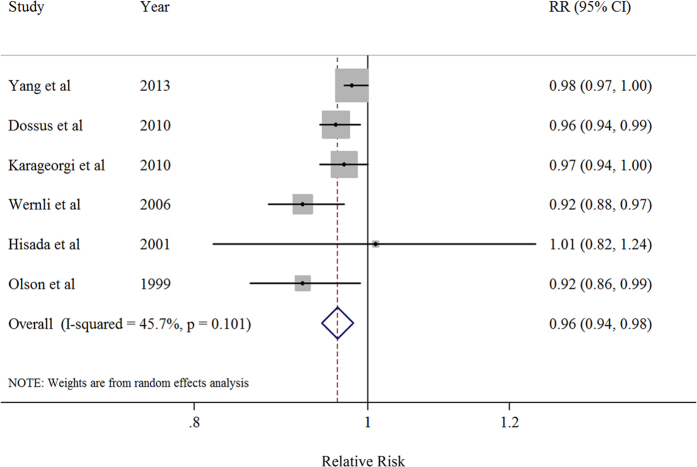
Dose-response analysis (random effects model) between per 2 year delay in menarcheal age and risk of endometrial cancer. Squares indicate study-specific relative risks (size of the square reflects the study-specific statistical weight); horizontal lines indicate 95% CIs; diamond indicates the summary relative risk estimate with its 95% CI. CI: confidence interval; RR: relative risk.

Six studies were included in the dose-response analysis^9^,^21^,^28^,^29^,^30^,^33^. There was an 4% reduction in risk for per 2 years delay in menarcheal age (summarized RR = 0.96; 95% CI = 0.94–0.98) with moderate but non-significant heterogeneity (*I*^2^ = 45.7%, *P* = 0.101) (Table 3, Fig. 3). There was no indication of a publication bias by using Egger’s test (*P*-bias = 0.152) and no asymmetry was observed in funnel plots when inspected visually. There was no evidence of a nonlinear association between the menarcheal age and endometrial cancer risk (*P* for nonlinearity = 0.16).

### Subgroup analyses

In subgroup analyses, possible differences between risk estimates by various study characteristics were examined. The finding of decreased endometrial cancer risk with later menarcheal age was consistently observed in almost all of the stratified analyses, although not all strata showed statistical significance. Furthermore, there was no evidence of significant heterogeneity between subgroups detected by meta-regression analyses (Table 2 and Table 3). When stratified by whether considering or adjustment for potential confounders, we did not find a significant difference between estimates adjusted and those not adjusted for specific factors (Table 2). Likewise, similar results were also observed in the dose-response analysis (Table 3).

### Sensitivity analyses

In a sensitivity analysis, we sequentially removed one study at a time and re-analyzed the data. The 8 study-specific RRs of the oldest *versus* youngest menarcheal age ranged from a low of 0.65 (95% CI: 0.53–0.79, *I*^2^ = 40.8%, *P* = 0.119) after omission of the study by Yang *et al.*^28^ to a high of 0.74 (95% CI: 0.66–0.83, *I*^2^ = 0%, *P* = 0.461) after omission of the study by Wernli *et al.*^9^. Similar analyses were also carried out in the dose-response analysis, with study specific RRs ranged from a low of 0.95 (95% CI: 0.93–0.97, *I*^2^ = 15.7%, *P* = 0.314) after omission of the study by Yang *et al.*^28^ to a high of 0.97 (95% CI: 0.96–0.99, *I*^2^ = 10.7%, *P* = 0.345) after omission of the study by Wernli *et al.*^9^.

## Discussion

To the best of our knowledge, this is the first comprehensive and quantitative assessment to explore the relationship between menarcheal age and risk of endometrial cancer. Overall, menarcheal age was found to be associated with an estimated 32% reduction in endometrial cancer risk when the oldest menarcheal age category compared to the youngest. Additionally, the results of dose-response analyses suggested an 4% reduction in endometrial cancer risk for per 2 years delay in menarcheal age (summarized RR = 0.96, 95% CI: 0.94–0.98). These significant results were consistent within subgroup analyses and across sensitivity analyses.

The association between hormonal risk factors and endometrial cancer risk could be partly attributed to the “unopposed estrogen” hypothesis. This hypothesis proposes that exposure to estrogen, in the absence of sufficient progestin, leads to increase mitotic activity, DNA replication, and somatic mutations of endometrial cells that may result in malignant transformations^2^,^3^,^4^,^5^,^34^,^35^. In addition, as the beginning of the menstruation span, later menarcheal age might decrease the risk of endometrial cancer by decreasing a woman’s lifetime number of ovulations which was characterized by exposure to endogenous estrogen and progesterone deficiency. Several epidemiologic studies^8^,^9^,^20^,^21^,^23^,^35^ have reported that longer year of menstruation span significantly increased the risk of endometrial cancer, which partly supported the findings in this meta-analysis, as well as the hypothesis which suggested that endometrial cancer risk might related to the lifetime number of ovulations. A recent study^36^, the Epidemiology of Endometrial Cancer Consortium (E2C2), has pooled of 15 observational studies (10 prospective and 5 case-control studies), provided evidence that older menarcheal age was significant inverse associated with endometrioid endometrial carcinoma (≥15 versus. <11 menarcheal age, OR: 0.63, 95% CI: 0.54–0.72, *P* trend = 0.04), which partly supported the findings of this meta-analysis. However, none of the included studies of this meta-analysis demonstrated the information of different histological type which limited us to confirm their finding. On the other hand, Type I endometrial cancers are mostly endometrioid adenocarcinomas, which seem to develop from abnormal glandular proliferations (i.e., endometrial hyperplasia) driven by hormonal mechanisms. In contrast, Type II endometrial cancers often display serous or clear cell histology and arise from atrophic endometrium in a less hormonally dependent manner. In addition, subtypes of these cancers are characterized by distinctive molecular alterations, and endometrioid carcinomas are more clearly linked to elevated levels of sex-steroid hormones and expression of hormone receptors^28^,^37^,^38^. However, only Yang *et al.*^28^ divided the main analyses by the subtype of endometrial cancer. Given this, further studies are warranted to focus on this issue. In our previous meta-analysis of menarcheal age and ovarian cancer risk^11^, we found that the categories used for the age at menarche varied from studies. Although the results of meta-regression of our study found no evidence for heterogeneity of different study populations, each population was unique and the category of menarcheal age might different from other studies. For example, Wernli *et al.*^9^ reported that the youngest and oldest category of menarcheal age in a cohort study with 267,400 women which was carried out in China was less than 13 years and over than 17 years, respectively. By comparison, Olson *et al.* reported that the youngest and oldest category of menarcheal age in a cohort study with 24,848 women which was carried out in Iowa was less than 11 years and over than 14 years, respectively. Since this, the interpretation of the results when using the oldest category of menarcheal age compared to the youngest should be cautiously. However, the results of dose-response analysis which could reduce the impact from the different category of menarcheal age of these included studies were in accordance with the results of oldest *versus* youngest category analysis which not only demonstrated the findings of this meta-analysis were robust but suggested that this aforementioned association was independent of the category methods. On the other hand, several genome-wide association studies (GWAS) have identified that genetic factors may associated with menarcheal age^39^,^40^,^41^,^42^ which also might attributed to the different category of menarcheal age. Therefore, given six prospective studies were included in the dose-response analysis of this study, more epidemiologic studies should provide the risk estimates which modeling a one or two-year increment in menarcheal age continuously, as well as focus on whether genetic polymorphisms may modify the association between menarcheal age and endometrial cancer risk.

A strength of this study is that it included a relatively larger sample sizes than any other individual studies with a total of 4553 endometrial cancer cases and approximately 949,945 non-cases. Thus, we had statistical power to detect moderate and weak associations and carry out subgroup analyses by potential confounding factors and study characteristics. Another important strength of this meta-analysis was that we included all the prospective design studies and the results are unlikely to be explained by the bias of traditional retrospective studies.

This study, however, also has limitations. First, since all included prospective studies were based on the observational design, it is possible that these aforementioned observed significant inverse associations could be due to the unmeasured or residual confounding. Many factors known to affect endometrial cancer risk, especially BMI^12^,^43^ is either associated with menarcheal age or as an important risk factor for endometrial cancer. However, since we could not get the access of raw data of each included studies and none of them have clarified the detail information of potential confounders in their published articles, thus we carried out the stratified analyses by these potential confounders which were adjusted in the multivariable models of their primary analyses. On the other hand, cohort studies more likely to present the age at menarche or other important risk factors of endometrial cancer by the distribution of exposure instead of by case status, which might partly results in limited studies included in this meta-analysis. Although numerous subgroup analyses were carries out and the results generally showed inverse associations, the limitation by the numbers of included prospective studies still should be considered. Second, because of the majority of the prospective study included the participants later than the focused exposure happened, thus females included in this study generally reported their exposure information long after menarche which may result in potential information bias. However, given that menarche is the milestone of puberty initiation and Bean *et al.*^44^ previously reported that age at menarche has reasonably good recall accuracy. Therefore, this kind of information bias could be limited in our study. Third, to our knowledge, the category of menarcheal age differed between studies and may have contributed to the heterogeneity in results. However, few of the included studies reported how they categorized the menarcheal age, and thus, we hardly considered this point in the subgroup analysis and ruled out the heterogeneity thoroughly. Furthermore, publication bias can also be a problem in meta-analyses of published studies but we found no statistical evidence of publication bias in this meta-analysis. Finally, as the starting point of a woman’s menstrual history, menarcheal age only focused on the early period of menstrual history, future studies should provide more evidence of the reproductive factors of other periods.

In summary, this meta-analysis suggested that later menarcheal age was inversely associated with the risk of endometrial cancer. Further studies, especially large consortium or pooled studies would be of interest to fully adjust for potential confounders and distinguish whether other risk factors which closely associated with both menstruation span and hormone change (e.g., age at menopause and multiparity) might have the similar inverse association with endometrial cancer risk and whether these associations might differ by the histological subtypes of endometrial cancer.

## Methods

### Literature search

We performed a comprehensive literature search to the end of June 2015 using the MEDLINE (PubMed) and EMBASE databases limited to the studies of humans by using the following search key words and Medical Subject Headings terms: (menarche OR reproductive factors OR reproduction) AND (endometrial OR endometrium OR corpus uteri OR uterine corpus) AND (cancer OR neoplasm OR carcinoma OR tumor). Additionally, the reference lists of retrieved articles were also scrutinized for additional studies by manual search. A similar search strategy was utilized for our previous meta-analysis of menarcheal age and ovarian cancer^11^. This study was planned, conducted, and reported in adherence to standards of quality for reporting meta-analyses^45^.

### Study selection criteria

Published studies were included by the following selection criteria if they 1) used a prospective study design, including cohort, case-cohort, and nested case-control studies; 2) evaluated the association between menarcheal age and incident endometrial cancer risk; and 3) presented relative risk (RR) or hazard ratio (HR) estimates with 95% confidence intervals (CI), standard errors (SE) or data necessary to calculate these.

Published manuscripts were excluded by the following exclusion criteria if they 1) had a retrospective design; 2) the estimates were presented without SE or other information that allowed calculation of SE; 3) reported exclusively on endometrial cancer mortality. When multiple publications from the same study were available, we used the publication with the largest number of cases and most applicable information.

All above study selection and exclusion procedures were carried out by two independent investigators (T-TG and X-XM).

### Data abstraction and quality assessment

For each eligible study, two investigators (T-TG and X-XM) independently performed the eligibility evaluation and data abstraction. Discrepancies were settled by consensus or by involving a third reviewer (Y-LW) for adjudication. Data abstracted from each study were: author list, year of publication, country where the study was performed, study design, study sample size (number of cases and cohort size), range of follow-up for cohort studies, exposure and outcome assessment and menarcheal age categories, study-specific adjusted RRs or HRs with their 95% CIs for the oldest *versus* youngest category of menarcheal age (we extracted the RRs that reflected the greatest degree of control for potential confounders for use in the main analyses), and factors matched between cases and controls in nested case-control study and potential confounders adjusted in the data analysis.

Since all included studies are prospective study and similar to our previous studies^11^,^46^, we did not assign quality scores^47^ to assess the methodological quality of all the included studies which lacks demonstrated validity, and sometimes results may not be associated with quality^48^, but investigated whether specific study characteristics, such as study design and adjustment for confounders, which are indicators of study quality, influenced the results in subgroup analyses.

### Statistical analysis

The study-specific adjusted RRs were used as the common measure of association across studies. For the meta-analysis, we assumed that estimates of risk, rate or hazard ratios from prospective studies were all valid estimates of the RR and we therefore report all results as the RR for simplicity. For study of Yang *et al.*^28^ that reported results separately of type I and type II endometrial cancer, but not combined, we pooled the results using a fixed-effect model to obtain an overall combined estimate before combining with the rest of the studies.

The possible heterogeneity in results across studies was examined by using the *I*^2^ statistics^49^. The *I*^2^ statistic represents the proportion of total variation contributed by between-study variation^49^. We used a random-effects model to calculate summary RR of endometrial cancer with 95% CIs, considering within- and between-study variation^50^. Heterogeneity between subgroups was evaluated by meta-regression^46^.

For the dose-response analysis, we used the generalized least-squares trend estimation method developed by Greenland *et al.*^51^ and Orsini *et al.*^52^ to compute study-specific slopes (linear trends) and 95% CIs from the natural logs of the RRs and CIs across categories of the menarcheal age. The aforementioned method requires the following information: 1) the distribution of cases and person-years or non-cases and the RRs with the variance estimates for at least three quantitative exposure categories; 2) the median or mean level of these exposures in each category (if reported by ranges, mean level were calculated by averaging the lower and upper bound; if the lowest category was open ended, the lowest boundary was considered to be zero; if the highest category was open ended, the open-ended interval length was assumed to be the same as the adjacent interval). Since these criteria, six studies^9^,^21^,^28^,^29^,^30^,^33^ were included in the dose-response analysis of menarcheal age and endometrial cancer risk. Furthermore, a potential nonlinear dose-response relationship between the menarcheal age and endometrial cancer risk was modeled by using restricted cubic splines with 3 knots at fixed percentiles (10%, 50%, and 90%) of the distribution of exposure. We calculated an overall *P* value by testing that these two regression coefficients were simultaneously equal to zero. We calculated a *P* value for nonlinearity by testing that the coefficient of the second spline was equal to zero. The details of this method has been published elsewhere^53^,^54^. The dose-response results are presented for a two years delay in menarcheal age.

To investigate possible sources of heterogeneity of main results, we carried out stratified analyses by the following study features: duration of follow-up (<10 *versus* ≥10 years), number of cases (<400 *versus* ≥400), method of exposure assessment (self-administered questionnaire *versus* trained interviewers), study population (American *versus* Non-American), and factors matched between cases and controls in nested case-control study and potential confounders adjusted in the data analyses (body mass index, parity, oral contraceptive use, exogenous hormones use, menopause status, and smoking status). Finally, sensitivity analysis was executed by deleting each study in turn to reflect the influence of individual data set on the overall estimate.

Small study bias, such as publication bias, was evaluated by Egger’s linear regression^55^ and funnel plots. A *P*-value less than 0.05 for Egger’s test was considered representative of significant statistical publication bias. Statistical analyses were performed with Stata (version 11.2; StataCorp, College Station, TX). *P*-values were two sided with a significance level of 0.05.

## Additional Information

**How to cite this article**: Gong, T.-T. *et al.* Age at menarche and endometrial cancer risk: a dose-response meta-analysis of prospective studies. *Sci. Rep.* **5**, 14051; doi: 10.1038/srep14051 (2015).

## Figures and Tables

**Table 1:** Characteristics of included studies of menarcheal age and endometrial cancer risk.

**First author, publication year [reference], Country, Study design**	**Cases/subject (age), duration of follow up**	**Source of cases**	**Menarcheal age categories (exposure assessment)**	**RR/HR (95%CI)**	**Matched/Adjusted factors**
Yang *et al.*^28^, 2013, USA, CS	1450/114,409 (50–69y), 9.4y	Cancer registry	<13	1.00 (ref)	Age, OC use, menopausal hormone therapy use, parity, BMI, age at menopause, race, and smoking status
			13–14	0.91 (0.82–1.02)	
			≥15 (Self-administered questionnaire)	0.80 (0.65–0.97)	
Dossus *et al.*^21^, 2010, Europe, CS	1017/301,601 (35–70y), 8.7y	Cancer registry	<12	1.00 (ref)	Age, center, BMI, physical activity, alcohol, diabetes, smoking status and education
			12	0.97 (0.80–1.19)	
			13	0.78 (0.63–0.95)	
			14	0.80 (0.65–0.98)	
			≥15 (Self-administered questionnaire)	0.72 (0.58–0.90)	
Karageorgi *et al.*^29^, 2010, USA, CS	778/121,700 (30–55y), 28y	Medical records	≤11	1.00 (ref)	Age, parity, age at first birth, age at last birth, OC duration, postmenopausal hormone use duration, BMI, smoking, diabetes, family history of endometrial cancer and age at menopause
			12	0.99 (0.81–1.20)	
			13	0.80 (0.66–0.98)	
			14	0.90 (0.70–1.16)	
			≥15 (Self-administered questionnaire)	0.76 (0.55–1.04)	
Setiawan *et al.*^31^, 2007, USA, CS	321/46,933 (45–75y), 7.3y	Cancer registry	≤12	1.00 (ref)	Race/ethnicity, BMI, age at natural menopause, parity, hormone therapy use, OC use, smoking, diabetes, hypertension, family history of endometrial cancer
			13–14	0.86 (0.68–1.10)	
			≥15 (Self-administered questionnaire)	0.85 (0.60–1.22)	
Wernli *et al.*^9^, 2006, China, CS	206/267,400 (≥30y), 10y	Medical records	≤13	1.00 (ref)	Age and parity number
			14	0.48 (0.28–0.82)	
			15	0.73 (0.47–1.16)	
			16	0.77 (0.49–1.20)	
			≥17 (Self-administered questionnaire)	0.40 (0.25–0.63)	
Hisada *et al.*^33^, 2001†, USA, NC-CS	39/194/15,528 (N/A), 27y	Cancer registry	≤11	1.00 (ref)	Age and race
			12	0.89 (0.18–4.30)	
			13	1.44 (0.34–6.09)	
			≥14 (Trained interviewer)	0.67 (0.10–4.33)	
Olson *et al.*^30^, 1999, USA, CS	322/24,848 (55–69y), 10y	Cancer registry	≤11	1.00 (ref)	Age
			11–12	0.7 (0.5–1.2)	
			12–13	0.6 (0.4–1.0)	
			>14 (Self-administered questionnaire)	0.4 (0.2–0.8)	
Kvale *et al.*^32^, 1988, Norway, CS	420/62,079 (27–69y), 20y	Medical records	≤12	1.00 (ref)	Age at start of follow-up, urban/rural place of residence, and parity
			13	N/A	
			14	N/A	
			15	N/A	
			16	N/A	
			≥17 (Self-administered questionnaire)	0.58 (0.39–0.88)	

RR: relative risk; CI: confidence interval; CS: cohort study; NC-CS: nested case-control study; N/A: not available; BMI: body mass index; OC: oral contraceptive.

^†^Odds ratio and 95% CI calculated from published data using EpiCalc 2000.

**Table 2:** Summary risk estimates of the association between menarcheal age and endometrial cancer risk, oldest *versus* youngest category.

	**No. of**	**Summary RR**	***I*^2^**	*P*_h_^*^	*P*_h_^**^
	**studies**	**(95% CI)**	**Value (%)**		
**Overall**	8	0.68 (0.58–0.81)	41.9	0.099	—
Subgroup analyses					
Duration of follow-up					0.085
<10y	3	0.78 (0.68–0.89)	0	0.674	
≥10y	5	0.56 (0.42–0.74)	37.2	0.173	
Number of cases					0.281
<400	4	0.74 (0.65–0.84)	62.4	0.046	
≥400	4	0.55 (0.33–0.90)	0	0.563	
Exposure Assessment					0.992
Trained interviewer	1	1.01 (0.82–1.24)	N/A	N/A	
Self-administered questionnaire	7	0.68 (0.57–0.81)	50.2	0.061	
Study population					0.232
Non-American	3	0.58 (0.41–0.81)	62.2	0.071	
American	5	0.77 (0.67–0.90)	0	0.421	
Adjustment for confounders or important risk factors					
Body mass index					0.057
Yes	4	0.77 (0.68–0.88)	0	0.849	
No	4	0.48 (0.36–0.63)	0	0.612	
Parity					0.710
Yes	5	0.69 (0.55–0.86)	57.0	0.054	
No	3	0.64 (0.45–0.91)	20.3	0.285	
Oral contraceptive use					0.085
Yes	3	0.80 (0.69–0.93)	0	0.900	
No	5	0.56 (0.42–0.74)	41.8	0.143	
Exogenous hormones use					0.085
Yes	3	0.80 (0.69–0.93)	0	0.900	
No	5	0.56 (0.42–0.74)	41.8	0.143	
Menopause status					0.085
Yes	3	0.80 (0.69–0.93)	0	0.900	
No	5	0.56 (0.42–0.74)	41.8	0.143	
Smoking status					0.057
Yes	4	0.77 (0.68–0.88)	0	0.849	
No	4	0.48 (0.36–0.63)	0	0.612	

RR: relative risk; CI: confidence interval; N/A: not available.

^*^P value for heterogeneity within each subgroup.

^**^P value for heterogeneity between subgroups with meta-regression analysis.

**Table 3:** Summary risk estimates of the association between menarcheal age and endometrial cancer risk, dose-response analysis of per 2 year delay.

	**No. of**	**Summary RR**	***I*^2^**	*P*_h_^*^	*P*_h_^**^
	**studies**	(**95% CI)**	**Value** (**%)**		
Overall	6	0.96 (0.94–0.98)	45.7	0.101	—
Subgroup analyses					
Duration of follow-up					0.303
<10y	2	0.97 (0.95–0.99)	44.7	0.179	
≥10y	4	0.95 (0.91–0.98)	33.7	0.210	
Number of cases					0.067
<400	3	0.92 (0.89–0.96)	0	0.868	
≥400	3	0.97 (0.96–0.99)	0	0.388	
Exposure Assessment					0.680
Trained interviewer	1	1.01 (0.82–1.24)	N/A	N/A	
Self-administered questionnaire	6	0.96 (0.94–0.98)	55.8	0.066	
Study population					0.259
Non-American	2	0.95 (0.91–0.98)	56.3	0.130	
American	4	0.98 (0.96–0.99)	7.5	0.356	
Adjustment for confounders or important risk factors					
Body mass index					0.067
Yes	3	0.97 (0.96–0.99)	0	0.388	
No	3	0.92 (0.89–0.96)	0	0.868	
Parity					0.652
Yes	3	0.96 (0.94–0.99)	66.5	0.051	
No	3	0.96 (0.93–0.98)	0	0.469	
Oral contraceptive use					0.080
Yes	2	0.98 (0.97–0.99)	0	0.560	
No	4	0.95 (0.92–0.97)	12.2	0.332	
Exogenous hormones use					0.080
Yes	2	0.98 (0.97–0.99)	0	0.560	
No	4	0.95 (0.92–0.97)	12.2	0.332	
Menopause status					0.080
Yes	2	0.98 (0.97–0.99)	0	0.560	
No	4	0.95 (0.92–0.97)	12.2	0.332	
Smoking status					0.067
Yes	3	0.97 (0.96–0.99)	0	0.388	
No	3	0.92 (0.89–0.96)	0	0.868	

RR: relative risk; CI: confidence interval; N/A: not available.

^*^P value for heterogeneity within each subgroup.

^**^P value for heterogeneity between subgroups with meta-regression analysis.

## References

[b1] Jemal A. *et al.* Global cancer statistics. CA Cancer J Clin 61, 69–90 (2011). 21296855 10.3322/caac.20107

[b2] Cook L. S., Weiss N. S., Doherty J. A. & Chen C. Endometrial Cancer. In: Cancer epidemiology and prevention. 3rd edn, (eds Schottenfeld D. & Fraumeni, Jr. ) 1027–1043. (Oxford University Press, 2006).

[b3] Key T. J. & Pike M. C. The dose-effect relationship between ‘unopposed’ oestrogens and endometrial mitotic rate: its central role in explaining and predicting endometrial cancer risk. Br J Cancer 57, 205–12 (1988). 3358913 10.1038/bjc.1988.44 PMC2246441

[b4] Ruddon R. W. Cancer Biology, 3rd edn, 240–251. (Oxford University Press, 1995).

[b5] Pitot H. C. Fundamentals of Oncology. (Marcel Dekker Inc, 1986).

[b6] King R. J. B. & Whitehead M. I. Estrogen and progestin effects on epithelium and stroma from pre- and postmenopausal endometria: application to clinical studies of the climacteric syndrome. In: Steroids and Endometrial Cancer. (eds Jasonni V. M. ) (New York Raven Press, 1983).

[b7] Akhmedkhanov A., Zeleniuch-Jacquotte A. & Toniolo P. Role of exogenous and endogenous hormones in endometrial cancer: review of the evidence and research perspectives. Ann N Y Acad Sci 943, 296–315 (2001). 11594550 10.1111/j.1749-6632.2001.tb03811.x

[b8] Pettersson B., Adami H. O., Bergstrom R. & Johansson E. D. Menstruation span—a time-limited risk factor for endometrial carcinoma. Acta Obstet Gynecol Scand 65, 247–55 (1986). 3739631 10.3109/00016348609155179

[b9] Wernli K. J. *et al.* Menstrual and reproductive factors in relation to risk of endometrial cancer in Chinese women. Cancer Causes Control 17, 949–55 (2006). 16841262 10.1007/s10552-006-0034-6

[b10] Li C. Y. *et al.* Age at menarche and risk of colorectal cancer: a meta-analysis. PLoS One 8, e65645 (2013). 23762403 10.1371/journal.pone.0065645 PMC3675201

[b11] Gong T. T. *et al.* Age at menarche and risk of ovarian cancer: a meta-analysis of epidemiological studies. Int J Cancer 132, 2894–900 (2013). 23175139 10.1002/ijc.27952 PMC3806278

[b12] Currie C. *et al.* Is obesity at individual and national level associated with lower age at menarche? Evidence from 34 countries in the Health Behaviour in School-aged Children Study. J Adolesc Health 50, 621–6 (2012). 22626490 10.1016/j.jadohealth.2011.10.254

[b13] Bralic I. *et al.* Association of early menarche age and overweight/obesity. J Pediatr Endocrinol Metab 25, 57–62 (2012). 22570951 10.1515/jpem-2011-0277

[b14] Mandel D. *et al.* Age at menarche and body mass index: a population study. J Pediatr Endocrinol Metab 17, 1507–10 (2004). 15570987 10.1515/jpem.2004.17.11.1507

[b15] Jacobsen B. K., Heuch I. & Kvale G. Association of low age at menarche with increased all-cause mortality: a 37-year follow-up of 61,319 Norwegian women. Am J Epidemiol 166, 1431–7 (2007). 17875585 10.1093/aje/kwm237

[b16] Tamakoshi K., Yatsuya H. & Tamakoshi A. Early age at menarche associated with increased all-cause mortality. Eur J Epidemiol 26, 771–8 (2011). 22006230 10.1007/s10654-011-9623-0

[b17] Wu X. *et al.* Age at menarche and natural menopause and number of reproductive years in association with mortality: results from a median follow-up of 11.2 years among 31,955 naturally menopausal Chinese women. PLoS One 9, e103673 (2014). 25090234 10.1371/journal.pone.0103673 PMC4121137

[b18] Mueller N. T. *et al.* Age at menarche and cardiovascular disease mortality in Singaporean Chinese women: the Singapore Chinese Health Study. Ann Epidemiol 22, 717–22 (2012). 22939833 10.1016/j.annepidem.2012.08.002 PMC3459135

[b19] Brinton L. A. *et al.* Reproductive, menstrual, and medical risk factors for endometrial cancer: results from a case-control study. Am J Obstet Gynecol 167, 1317–25 (1992). 1442985 10.1016/s0002-9378(11)91709-8

[b20] Xu W. H. *et al.* Menstrual and reproductive factors and endometrial cancer risk: Results from a population-based case-control study in urban Shanghai. Int J Cancer 108, 613–9 (2004). 14696129 10.1002/ijc.11598

[b21] Dossus L. *et al.* Reproductive risk factors and endometrial cancer: the European Prospective Investigation into Cancer and Nutrition. Int J Cancer 127, 442–51 (2010). 19924816 10.1002/ijc.25050

[b22] Wernli K. J. *et al.* Occupational risk factors for endometrial cancer among textile workers in Shanghai, China. Am J Ind Med 51, 673–9 (2008). 18626909 10.1002/ajim.20614 PMC2574926

[b23] McPherson C. P. *et al.* Reproductive factors and risk of endometrial cancer. The Iowa Women’s Health Study. Am J Epidemiol 143, 1195–202 (1996). 8651218 10.1093/oxfordjournals.aje.a008707

[b24] Kabat G. C. *et al.* Adult height in relation to risk of cancer in a cohort of Canadian women. Int J Cancer 132, 1125–32 (2013). 22753236 10.1002/ijc.27704

[b25] Khan M. *et al.* Risk of endometrial cancer mortality by ever-use of sex hormones and other factors in Japan. Asian Pac J Cancer Prev 7, 260–6 (2006). 16839220

[b26] Kasum C. M. *et al.* Whole grain intake and incident endometrial cancer: the Iowa Women’s Health Study. Nutr Cancer 39, 180–6 (2001). 11759278 10.1207/S15327914nc392_4

[b27] Bergkvist L., Persson I., Adami H. O. & Schairer C. Risk factors for breast and endometrial cancer in a cohort of women treated with menopausal oestrogens. Int J Epidemiol 17, 732–7 (1988). 3225079 10.1093/ije/17.4.732

[b28] Yang H. P. *et al.* Endometrial cancer risk factors by 2 main histologic subtypes: the NIH-AARP Diet and Health Study. Am J Epidemiol 177, 142–51 (2013). 23171881 10.1093/aje/kws200 PMC3590033

[b29] Karageorgi S., Hankinson S. E., Kraft P. & De Vivo I. Reproductive factors and postmenopausal hormone use in relation to endometrial cancer risk in the Nurses’ Health Study cohort 1976–2004. Int J Cancer 126, 208–16 (2010). 19551854 10.1002/ijc.24672 PMC2784268

[b30] Olson J. E., Sellers T. A., Anderson K. E. & Folsom A. R. Does a family history of cancer increase the risk for postmenopausal endometrial carcinoma? A prospective cohort study and a nested case-control family study of older women. Cancer 85, 2444–9 (1999). 10357416

[b31] Setiawan V. W. *et al.* Racial/ethnic differences in endometrial cancer risk: the multiethnic cohort study. Am J Epidemiol 165, 262–70 (2007). 17090617 10.1093/aje/kwk010

[b32] Kvale G., Heuch I. & Ursin G. Reproductive factors and risk of cancer of the uterine corpus: a prospective study. Cancer Res 48, 6217–21 (1988). 3167867

[b33] Hisada M. *et al.* Prospective study of antibody to human papilloma virus type 16 and risk of cervical, endometrial, and ovarian cancers (United States). Cancer Causes Control 12, 335–41 (2001). 11456229 10.1023/a:1011236803257

[b34] Parazzini F., La Vecchia C., Bocciolone L. & Franceschi S. The epidemiology of endometrial cancer. Gynecol Oncol 41, 1–16 (1991). 2026352 10.1016/0090-8258(91)90246-2

[b35] Zucchetto A. *et al.* Hormone-related factors and gynecological conditions in relation to endometrial cancer risk. Eur J Cancer Prev 18, 316–21 (2009). 19554665 10.1097/cej.0b013e328329d830

[b36] Felix A. S. *et al.* The etiology of uterine sarcomas: a pooled analysis of the epidemiology of endometrial cancer consortium. Br J Cancer 108, 727–34 (2013). 23348519 10.1038/bjc.2013.2 PMC3593566

[b37] Sherman M. E. *et al.* Risk factors and hormone levels in patients with serous and endometrioid uterine carcinomas. Mod Pathol 10, 963–8 (1997). 9346174

[b38] Lax S. F., Pizer E. S., Ronnett B. M. & Kurman R. J. Clear cell carcinoma of the endometrium is characterized by a distinctive profile of p53, Ki-67, estrogen, and progesterone receptor expression. Hum Pathol 29, 551–8 (1998). 9635673 10.1016/s0046-8177(98)80002-6

[b39] He C. *et al.* Genome-wide association studies identify loci associated with age at menarche and age at natural menopause. Nat Genet 41, 724–8 (2009). 19448621 10.1038/ng.385 PMC2888798

[b40] Ong K. K. *et al.* Genetic variation in LIN28B is associated with the timing of puberty. Nat Genet 41, 729–33 (2009). 19448623 10.1038/ng.382 PMC3000552

[b41] Liu Y. Z. *et al.* Genome-wide association analyses identify SPOCK as a key novel gene underlying age at menarche. PLoS Genet 5, e1000420 (2009). 19282985 10.1371/journal.pgen.1000420 PMC2652107

[b42] Sulem P. *et al.* Genome-wide association study identifies sequence variants on 6q21 associated with age at menarche. Nat Genet 41, 734–8 (2009). 19448622 10.1038/ng.383

[b43] Crosbie E. J. *et al.* Body mass index, hormone replacement therapy, and endometrial cancer risk: a meta-analysis. Cancer Epidemiol Biomarkers Prev 19, 3119–30 (2010). 21030602 10.1158/1055-9965.EPI-10-0832

[b44] Bean J. A. *et al.* Variations in the reporting of menstrual histories. Am J Epidemiol 109, 181–5 (1979). 425957 10.1093/oxfordjournals.aje.a112673

[b45] Stroup D. F. *et al.* Meta-analysis of observational studies in epidemiology: a proposal for reporting. Meta-analysis Of Observational Studies in Epidemiology (MOOSE) group. JAMA 283, 2008–12 (2000). 10789670 10.1001/jama.283.15.2008

[b46] Wu Q. J. *et al.* Cruciferous vegetables intake and the risk of colorectal cancer: a meta-analysis of observational studies. Ann Oncol 24, 1079–87 (2013). 23211939 10.1093/annonc/mds601 PMC3603442

[b47] Wells G. A. *et al.* The Newcastle-Ottawa Scale (NOS) for assessing the quality of nonrandomised studies in meta-analyses. Available from: http://www.ohri.ca/programs/clinical_epidemiology/oxford.asp. (Date of access: 10/March/2015).

[b48] Greenland S. Invited commentary: a critical look at some popular meta-analytic methods. Am J Epidemiol 140, 290–6 (1994). 8030632 10.1093/oxfordjournals.aje.a117248

[b49] Higgins J. P. & Thompson S. G. Quantifying heterogeneity in a meta-analysis. Stat Med 21, 1539–58 (2002). 12111919 10.1002/sim.1186

[b50] DerSimonian R. & Laird N. Meta-analysis in clinical trials. Control Clin Trials 7, 177–88 (1986). 3802833 10.1016/0197-2456(86)90046-2

[b51] Greenland S. & Longnecker M. P. Methods for trend estimation from summarized dose-response data, with applications to meta-analysis. Am J Epidemiol 135, 1301–9 (1992). 1626547 10.1093/oxfordjournals.aje.a116237

[b52] Orsini N. *et al.* Meta-analysis for linear and nonlinear dose-response relations: examples, an evaluation of approximations, and software. Am J Epidemiol 175, 66–73 (2012). 22135359 10.1093/aje/kwr265 PMC3244608

[b53] Royston P. A strategy for modelling the effect of a continuous covariate in medicine and epidemiology. Stat Med 19, 1831–47 (2000). 10867674 10.1002/1097-0258(20000730)19:14<1831::aid-sim502>3.0.co;2-1

[b54] Bagnardi V., Zambon A., Quatto P. & Corrao G. Flexible meta-regression functions for modeling aggregate dose-response data, with an application to alcohol and mortality. Am J Epidemiol 159, 1077–86 (2004). 15155292 10.1093/aje/kwh142

[b55] Egger M., Davey S. G., Schneider M. & Minder C. Bias in meta-analysis detected by a simple, graphical test. BMJ 315, 629–34 (1997). 9310563 10.1136/bmj.315.7109.629 PMC2127453

